# A Painless Restricted Motion of the Thumb: What Etiology? About An Uncommon Tumor in Uncommon Localization

**DOI:** 10.1155/2020/5649204

**Published:** 2020-05-30

**Authors:** Naoufal Elghoul, Mohammed Benchakroun, Azzelarab Bennis, Omar Zaddoug, Ali Zine, Mansour Tanane, Abdeloihab Jaafar

**Affiliations:** Department of Orthopedic Surgery and Traumatology, Military Hospital Mohammed V (HMIMV), Faculty of Medicine and Pharmacy, University Mohammed V, BP 10100 Rabat, Morocco

## Abstract

Lipomas in fingers are rare and account for less than 1% of all cases. As a type of lipoma, the spindle cell lipoma is exceptional and it presents 1.5% of total adipocyte tumors. Moreover, its localization in the thumb is extremely rare. Only three cases have already been reported in adults; our case constitutes the fourth case, which is about a 61-year-old female who presented since 18 months a mass on the ulnar lateral aspect of the thumb. After clinical and radiological assessments, an entire excisional biopsy of the mass was performed. The histopathological analysis confirmed the spindle cell lipoma of the thumb. At the last follow-up of two years, the patient did well with no recurrence and no restricted motion of the thumb. So, although lipomas of the digit are rare, they should be considered a possible etiology of either painful or mechanic restricted motion of the digit.

## 1. Introduction

Lipomas account for approximately 16% of soft tissue mesenchymal tumors. However, they are rare in the hand and extremely rare in fingers that account for less than 1% of all cases [1]. Spindle cell lipoma is a type of lipoma that presents 1.5% of total adipocyte tumors [2]. The index and middle fingers were most commonly involved [3]. Therefore, its localization in the thumb is extremely rare [3–5]. Herein, we report a rare case of a variant of lipoma localized in the thumb.

## 2. Case Presentation

**Figure 1:**
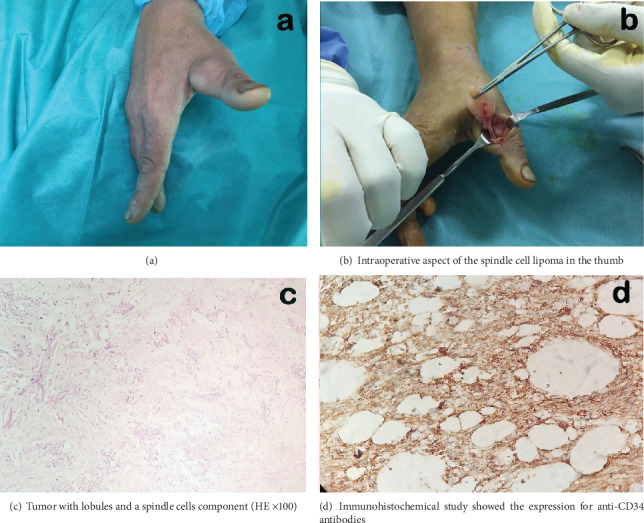


A 61-year-old female, maid, with no disease history, presented, since 18 months, a painless mass in the right thumb which increased slowly. The patient did not consult since it is not painful and does not bother her during physical activity. However, for the last two months, the mass started to bother her in her daily activities and it became a protruding mass; she consulted our orthopedic department. On admission, she was at good general health with no weight loss. At this visit, she reported a trauma history of the thumb two years ago. The clinical examination found a prominent mobile mass on the ulnar lateral aspect of the thumb with neither inflammatory changes nor a palpable pulse (Figure 1(a)). Passive mobility of the thumb was conserved. The neurovascular exam was normal. The plain anteroposterior radiograph of the digit was normal with no bone invasion. The ultrasound revealed a well-uniform low-echo mass measuring 45 × 30 × 20 mm on the ulnar lateral aspect of the thumb with no internal flow on Doppler, to be completed by magnetic resonance imaging for lesion characterization. Considering the patient's financial conditions, the magnetic resonance imaging was not performed. A few days later, the patient underwent surgery in which we performed an excisional biopsy of the mass of the thumb (Figure 1(b)). The postoperative was uneventful. One week later, the histopathological analysis showed a spindle cell lipoma of the thumb (Figures 1(c) and 1(d)). At the last follow-up of two years, the patient did well with no recurrence of the tumor and no restricted motion of the thumb.

## 3. Discussion

The lipomas of the digit are rare. Furthermore, as a type of lipomas, the spindle cell lipoma is a rare tumor that is reported for the first time by Enzinger and Harvey in 1975 [6]. Its localization in the thumb is exceptional, and only three cases were reported. The first and second cases were two males with this tumor on the palmar aspect of the thumb. The third case was a female with a small tumor of the thumb (10 × 10 mm). Therefore, our case was a female with spindle cell lipoma on the lateral aspect of the thumb measuring 45 × 30 mm that restricted its motion. To our knowledge, this is the fourth reported case of spindle cell lipoma of the thumb in adults, and it is the second case in a woman (Table 1). The etiology and pathogenesis of this tumor are still undetermined. However, a history of trauma was found in almost cases. Also, in our case, a history of trauma of the thumb was reported before the appearance of the tumor. The differential diagnoses are important to know which are the well-differentiated sclerosing liposarcoma and the spindle cell liposarcoma [7]. Given that the spindle cell lipoma may be misdiagnosed with liposarcoma in this location, it should be kept in mind that it could appear also in this site. Clinically, most lipomas are asymptomatic.

However, patients are often uncomfortable with the limitation of mobility once they reach a significant size [8] as in our case. Imaging studies (computed tomography scan and magnetic resonance imaging) appear to be diagnostic in 71% of the cases [9]. In our patient, only the ultrasound imaging was performed and it did not give a specific diagnosis. Surely the realization of magnetic resonance imaging could have helped us to characterize the lesion, but we were limited because the patient could not afford its expensive charge. Given the strong expression for CD34 in the immunohistochemical study, it confirmed for us the final diagnosis of spindle cell lipoma of the thumb. The treatment method for this tumor is marginal excision, together with the surrounding thin fibrous capsule [5].

## 4. Conclusion

Although lipomas of the digit are rare, they should always be kept in the list of the etiology of either painful or mechanic restricted motion of the digit. Besides, the spindle cell lipoma should be differentiated from the well-differentiated sclerosing liposarcoma and the spindle cell liposarcoma.

## Figures and Tables

**Table 1:** The four cases of spindle cell lipoma in adults.

Authors	Diagnosis	Localization	Age (year)	Gender
El Rayes et al. [3]	Spindle cell lipoma	Thumb	61	Male
Bhat et al. [4]	Spindle cell lipoma	Thumb	30	Female
Ebisudani et al. [5]	Spindle cell lipoma	Thumb	34	Male
Our case	Spindle cell lipoma	Thumb	61	Female
